# Clinical and Genetic Characterization of Craniosynostosis in Saudi Arabia

**DOI:** 10.3389/fped.2021.582816

**Published:** 2021-04-16

**Authors:** Malak Alghamdi, Taghreed R. Alhumsi, Ikhlass Altweijri, Waleed H. Alkhamis, Omar Barasain, Kelly J. Cardona-Londoño, Reshmi Ramakrishnan, Francisco J. Guzmán-Vega, Stefan T. Arold, Ghaida Ali, Nouran Adly, Hebatallah Ali, Ahmed Basudan, Muhammed A. Bakhrebah

**Affiliations:** ^1^Medical Genetic Division, Department of Pediatrics, College of Medicine, King Saud University, Riyadh, Saudi Arabia; ^2^Department of Pediatrics, King Saud University Medical City, Riyadh, Saudi Arabia; ^3^Department of Plastic Surgery, King Saud University Medical City, Riyadh, Saudi Arabia; ^4^Department of Neurosurgery, King Saud University Medical City, Riyadh, Saudi Arabia; ^5^Obstetrics and Gynecology, King Saud University Medical City, Riyadh, Saudi Arabia; ^6^College of Medicine, King Saud University, Riyadh, Saudi Arabia; ^7^Biological and Environmental Science and Engineering (BESE), Computational Bioscience Research Center (CBRC), King Abdullah University of Science and Technology (KAUST), Thuwal, Saudi Arabia; ^8^Center de Biochimie Structurale, CNRS, INSERM, Université de Montpellier, Montpellier, France; ^9^College of Medicine, Imam Muhammad Ibn Saud University, Riyadh, Saudi Arabia; ^10^College of Medicine Research Centre, College of Medicine, King Saud University, Riyadh, Saudi Arabia; ^11^Chair of Medical and Molecular Genetics, Department of Clinical Laboratory Sciences, King Saud University, Riyadh, Saudi Arabia; ^12^Life Science and Environment Research Institute, King Abdulaziz City for Science and Technology, Riyadh, Saudi Arabia

**Keywords:** craniosynostosis, trigonocephaly, *TCF12* gene, exome sequencing, *FGFR2* gene, *ALPL* gene, *TWIST1* gene

## Abstract

**Background:** Craniosynostosis (CS) is defined as pre-mature fusion of one or more of the cranial sutures. CS is classified surgically as either simple or complex based on the number of cranial sutures involved. CS can also be classified genetically as isolated CS or syndromic CS if the patient has extracranial deformities. Currently, the link between clinical and genetic patterns of CS in the Saudi population is poorly understood.

**Methodology:** We conducted a retrospective cohort study among 28 CS patients, of which 24 were operated and four were not. Clinical and genetic data were collected between February 2015 and February 2019, from consenting patient's families. The electronic chart data were collected and analyzed including patient demographics, craniofacial features, other anomalies and dysmorphic features, operative data, intra cranial pressure (ICP), parent consanguinity and genetic testing results.

**Results:** The most common deformity in our population was trigonocephaly. The most performed procedure was cranial vault reconstruction with fronto-orbital advancement, followed by posterior vault distraction osteogenesis and suturectomy with barrel staving. Genetics analysis revealed pathogenic mutations in *FGFR2* (6 cases), *TWIST1* (3 cases), *ALPL* (2 cases), and *TCF12* (2 cases), and *FREM1* (2 case).

**Conclusion:** Compared to Western countries, our Saudi cohort displays significant differences in the prevalence of CS features, such as the types of sutures and prevalence of inherited CS. The genomic background allows our phenotype-genotype study to reclassify variants of unknown significance. Worldwide, the sagittal suture is the most commonly affected suture in simple CS, but in the Saudi population, the metopic suture fusion was most commonly seen in our clinic. Further studies are needed to investigate the characteristics of CS in our population in a multicenter setting.

## Introduction

Craniosynostosis (CS) is defined as pre-mature fusion of one or more of the cranial sutures. CS can be classified into non-syndromic CS (NSCS; also termed isolated CS) in which the deformity only involves the skull, or into syndromic CS (SCS), in which the deformity involves the skull and other extracranial deformities (1). The spectrum of CS is wide and complex. Ranging from single suture synostosis to multiple suture synostosis with extracranial manifestations in syndromic patients. The complexity of this condition usually requires a multidisciplinary team approach including a craniofacial surgeon, a pediatric neurosurgeon, a geneticist, an ophthalmologist, an otolaryngologist, and a social worker (2). The CS incidence shows geographic variations, but lies on average at one in 2,000–2,500 live births (1, 2). The variability in incidence is possibly due to differences in detection and reporting of the deformities due to their large variation in disease manifestation and origin, among other factors.

The initial diagnosis of craniosynostosis can be made clinically, and then be supported by a computerized tomography (CT) scan to reveal further details. Closure of each suture will cause a distinct skull shape. The metopic suture closure will cause a triangular skull shape, termed trigonocephaly. Closure of the sagittal suture will cause scaphocephaly (“boat-shaped” skull), whereas, brachycephaly (short skull) is caused by the closure of both coronal sutures. Plagiocephaly (bent skull shape) is usually caused by closure of either one of the coronal sutures or the lambdoid suture (3, 4). SCS typically involves deformities in more than one cranial suture, accompanied by extracranial manifestations. These patients usually have higher incidence of increased intracranial pressure (ICP) and decreased brain development (4). More than 200 syndromes have been linked to CS (5).

CS is inherited either in an autosomal dominant (AD) pattern, autosomal recessive (AR) pattern or as a sporadic inheritance. Many genes have been implicated in the inheritance of CS, whether isolated or syndromic. More than 50 genes have been identified as being linked to CS, along with genetic factors, epigenetic factors like microRNAs and mechanical forces that play a role in CS (6).

In our study, we describe the clinical and genetic characters of a cohort of CS 28 patients from Saudi Arabia. To our knowledge, this work represents the first assessment of the links between phenotype, clinical aspects and genetic cause of CS patients from this country, which has a unique genomic population makeup shaped by a high prevalence of consanguinity.

## Materials and Methods

### Study Population

**Figure 1:**
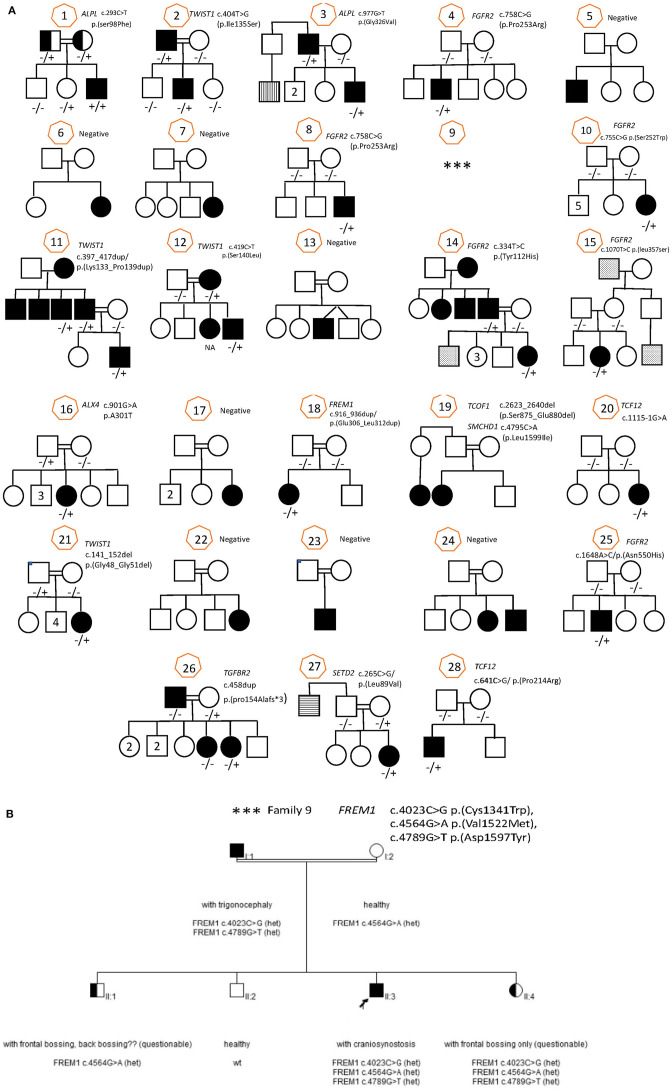
Family pedigree with sanger sequencing result for all family (28 families) including all informative family members. **(A)** Families 1–8, 10–28. **(B)** Family 9.

This single–center retrospective cohort study was conducted among 28 patients (see Figure 1 for pedigrees) with CS referred to the plastic surgery and pediatric departments in King Saud University Medical City, a tertiary referral center in Riyadh, Saudi Arabia, between February 2015 and February 2019. All patients who were diagnosed as CS were consented. A total of 24 patients were operated for craniosynostosis. Four patients were not operated, either due to patient refusal, operation in another center, or poor prognosis. Genetic testing was performed for all patients. The data collected from the electronic patient files included nationality, age at diagnosis, gender, type of suture fusion, shape of skull, increased ICP as evidenced by papilledema, dysmorphic features, age of surgery, type of surgery, genetic testing and mutations, antenatal course and parental consanguinity.

### Operative Techniques

**Figure 2:**
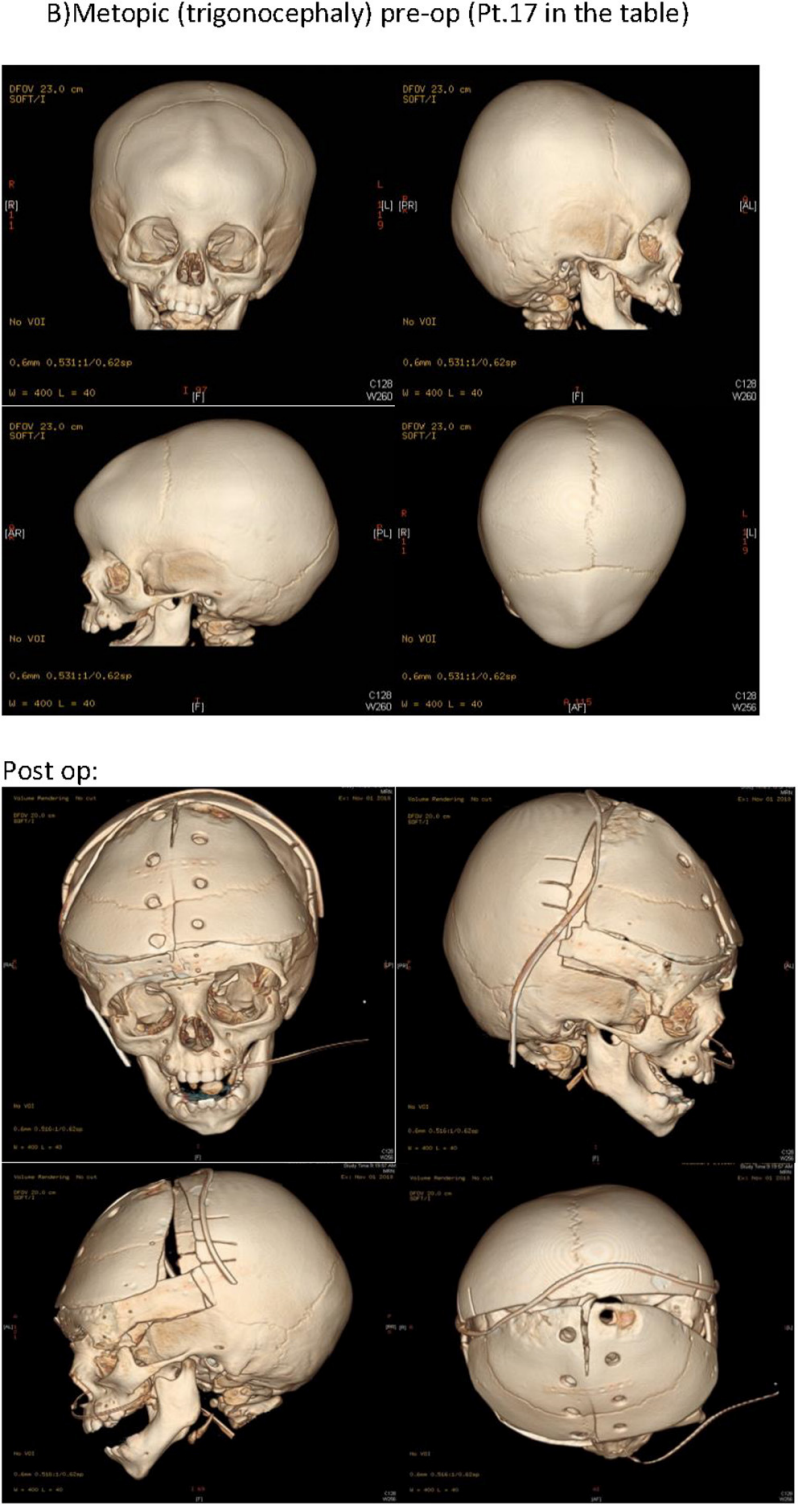
Pre-operative CT scan was done for all patients, along with 3D printed models to aid in patient explanation and decision making. Aperts: pre-op and post-op. CT (Pt.4 in the Table 1). B) Metopic (trigonocephaly) pre-op and post-op CT (Pt.17 in the Table 1).

The operative procedures performed included fronto-orbital bar advancement and anterior cranial vault reshaping (FOBA+CR), posterior cranial vault distraction osteogenesis (PVDO), and suturectomy with barrel staving. The procedure was chosen based on patient deformity (anterior or posterior), age, and need for expansion. Pre-operative CT scans were done for all patients, along with 3D printed models to aid in patient explanation and decision making (7) (Figure 2). Suturectomy and barrel staving was performed for two patients presenting with sagittal synostosis. This is a relatively short operative procedure and is performed in our center for children 4 months or younger as subsequent brain growth is responsible for post-operative skull molding in these children. The most common procedure performed in our center was FOBA+CR. This is a combined operative procedure performed by both a craniofacial surgeon and a neurosurgeon. All our patients received intra-operative blood transfusions, and tranexamic acid transfusion. Inion^®^ resorbable plates and screws were used for fixation of all reconstruction sites. Simple NSC patients were extubated, while SCS patients were usually kept intubated post-operatively until the next morning. All patients were sent to our pediatric intensive care unit overnight. The third procedure, PVDO, was performed on two patients in our study. The indication of such a procedure is reserved to older patients who have re-synostosed after an initial procedure done at due age. PVDO was used for these two cases because both presented with increased ICP.

### Molecular Testing

#### *FGFR2* Sanger Sequencing

DNA was amplified for the *FGFR2* gene and sequenced bidirectionally using an ABI 3730XL instrument. The data was analyzed against reference gene sequence and known variant position as requested.

#### Whole Exome Sequencing

DNA was extracted from dried blood spot in filter cards (Centocard)® using standard, spin column-based method. Approximately 37 Mb (214,405 exons) of the Consensus Coding Sequences (CCS) were enriched from fragmented genomic DNA by >340,000 probes that were designed against the human genome (Nextera Rapid Capture Exome, Illumina), and the generated library was sequenced on an Illumina NextSeq or HiSeq 4000 platform (Illumina) to an average coverage depth of 70–100X. An end-to-end inhouse bioinformatics pipeline, including base calling, primary filtering of low-quality reads and probable artifacts, and annotation of variants, was applied. All of the disease-causing variants that had been reported in HGMD®, ClinVar (class 1) as well as all variants with a minor allele frequency (MAF) of <1% in the ExAc database, were considered for this study. Our evaluation was focused on exons with intron boundaries ±20. All of the relevant inheritance patterns were considered, and family history and clinical information that were provided were used to evaluate the variants that we identified. Only the variants that were related to the phenotype are reported.

#### Re-classification of VOUS

According to the recommendation of the American College of Medical Genetics (ACMG) (8), variants of unknown significance (VOUS) were re-classified based on: phenotype–genotype (clinical) consistency, family segregation and the functional assays that were available in our clinical service demonstrated (Table 1). As shown in Table 1, functional assays were done for two patients: Patient #1 and Patient #3. Patient #1 was tested for alkaline phosphatase enzyme level to confirm pathogenicity of *ALPL* gene variants. Patient #3, who has a paternal inherited novel variant, and his father was tested for serum B6 level, urinary Phosphoethanolamine, and alkaline phosphatase to confirm clinical phenotype.

**Table 1:** Patient phenotype including clinical and molecular characteristics.

	**Demographic and surgical characteristics**						**Genetic characteristics**							
**#**	**Gender**	**Age at surgery**	**Suture closed**	**Papilledema**	Procedure^*^	**Consanguinity**	**Syndrome**	**Genetic test**	**Gene**	**Variant**	**Zygosity**	**Initial classification**	**Clinical consistency + Family segregation**	**Re-classification**
1	M	1y 6m	Multiple	No	FOBA + CR	No	Hypo-Phosphatasia	*WES*	*ALPL*	*c.293C>T p.(ser98Phe)*	*Homo*.	*Pathogenic*	-	-
2	M	1y 6m	Multiple	No	FOBA + CR	Yes	Seather chotzen	WES	*TWIST1*	c.404T>G (p.Ile135Ser)	Hetero.	VOUS	Inherited from affected father	Likely pathogenic
3	M	1y	Metopic	No	FOBA + CR	No	Hypo-Phosphatasia	WES	*ALPL*	c.977G>T p.(Gly326Val)	Hetero.	VOUS	[B6 (high), urinary PEA (high) and alkaline phosphatase (low)]	Likely pathogenic
4	M	11m	Bilateral Coronal	No	FOBA + CR	No	Apert	Direct Seq.	*FGFR2*	c.758C>G (p.Pro253Arg)	Hetero.	Pathogenic (*de novo*)	–	–
5	M	10m	Sagittal	Yes	FOBA + CR	No	–	WES	N	–	–	–	–	–
6	F	3m	Sagittal	No	Strip suturectomy + barrel staving	No	–	WES	N	–	–	–	–	–
7	F	4m	Sagittal	No	Strip suturectomy + barrel staving	No	–	WES	N	–	–	–	–	–
8	M	5m	Bilateral Coronal	Yes	FOBA + CR	No	Apert	Direct Seq.	*FGFR2*	c.758C>G (p.Pro253Arg)	Hetero	Pathogenic	–	–
9	M	1y 7m	Metopic	No	FOBA + CR	Yes	–	WES	*FREM1*	c.4023C>G p.(Cys1341Trp), c.4564G>A p.(Val1522Met), c.4789G>T p.(Asp1597Tyr)		VOUS	p.(Cys1341Trp) and p.(Asp1597Tyr) are in cis and likely segregate with the phenotype in this family.	Likely pathogenic
10	F	6m	Multiple	No	FOBA + CR	Yes	Apert	Direct Seq.	*FGFR2*	c.755C>G p.(Ser252Trp)	Hetero.	Pathogenic (*de novo*)	–	–
11	M	1y	Bilateral Coronal	Yes	FOBA + CR	Yes	Seather chotzen	WES	*TWIST1*	c.397_417dup/p. (Lys133_Pro139dup)	Hetero.	VOUS	Inherited from affected father	Likely Pathogenic
12	M	2y	Multiple	No	FOBA + CR	No	Seather chotzen	WES	*TWIST1*	c.419C>T/p.(Ser140Leu)	Hetero.	VOUS	Inherited from affected mother	Likely Pathogenic
13	M	8m	Bilateral Coronal	No	FOBA + CR	Yes	–	WES	N	–	–	–	–	–
14	F	1y 3m	Multiple	No	FOBA + CR	Yes	Pfeiffer syndrome -Type 1	Direct Seq.	*FGFR2*	c.334T>C p.(Tyr112His)	Heteroz.	Pathogenic/ Paternally inherited	–	–
15	M	6y	Multiple	Yes	PVDO	No	Crouzon	Direct Seq.	*FGFR2*	c.1070T>C p.(leu357ser)	Hetero.	Pathogenic	–	–
16	F	2y	Unilateral Coronal	Yes	FOBA + CR	Yes	Frontonasal dysplasia	WES	*ALX4*	c.901G>A:p.A301T	Hetero.	VOUS	The phenotype is not consistent with the genotype and the variant inherited from a healthy father	Likely benign
17	F	1y 2m	Metopic	No	FOBA + CR	No	-	WES	N	–	–	–	–	–
18	M	5y	Multiple	Yes	PVDO	No	-	WES	*FREM1*	c.916_936dup/ p.(Glu306_Leu312dup)	Hetero.	VOUS	A *de novo* variant	Likely pathogenic
19	F	7m	Multiple	No	FOBA + CR	Yes	-	WES	*TCOF1*	c.2623_2640del p.Ser875_Glu880del/	Hetero.	VOUS	Not consistent with the phenotype	Likely benign
									*SMCHD1*	c.4795C>T p.Leu1599Ile	Hetero.	VOUS	Not consistent with the phenotype	Likely benign
20	F	8 m	Unilateral Coronal	No	FOBA + CR	No	TCF related CS	WES	*TCF12*	c.1115-1G>A	Hetero.	VOUS	*de novo* variant and consistent with the phenotype	Likely pathogenic
21	F	2y 7m	Multiple	Yes	FOBA + CR	No	Seather chotzen	WES	*TWIST1*	c.141_152del p.(Gly48_Gly51del)	Hetero.	VOUS	Inherited from a healthy father	Likely benign
22	F	7m	Unilateral Coronal	No	FOBA + CR	No	–	WES	N	–	–	–	–	–
23	M	1y	Metopic	No	FOBA + CR	No	–	WES	N	–	–	–	–	–
24	M	6m	Metopic	No	FOBA + CR	No	–	WES	N	–	–	–	–	–
25	M	1y 6m	Multiple		None	No	Crouzon	Direct Seq.	*FGFR2*	c.1648A>C/ p.(Asn550His)	Hetero.	Pathogenic		
26	F	10m	Unilateral Coronal		None	Yes	Loeys-Dietz	WES	*TGFBR2*	c.458dup/ p.(pro154Alafs^*^3)	Hetero.	VOUS	Not consistent with the phenotype Inherited from a healthy mother	Likely Benign
27	F	7m	Metopic		None	No	Luscan-Lumish	WES	*SETD2*	c.265C>G/ p.(Leu89Val)	Hetero.	VOUS	Not consistent with the phenotype Inherited from a healthy mother	Likely Benign
28	M	2y	Sagittal		None	No	AD CR Type 3	WES	*TCF12*	c.641C>G/ p.(Pro214Arg)	Hetero.	VOUS	*de novo* variant and consistent with the phenotype	Likely pathogenic

*F, female; M, male; Y, years; M, months; FOBA+CR, fronto-orbital bar advancement and anterior cranial vault reshaping; WES, whole exome sequencing; Direct seq., FGFR2 gene sanger sequencing; Hetero, heterozygous; Homo, homozygous; VOUS, variant of unknown clinical significance*.

* *Mutation nomenclature*.

#### Bioinformatic Variant Assessment

Sequences were retrieved from the Uniprot database (9) Swiss-Model (10) and trRosetta (11) were used to produce structural models. RaptorX (12) was used to predict secondary structure and disorder, and the domain arrangement was based on PFAM (13). Conservation status of residues were predicted using Consurf (14). Models were manually inspected, and mutations evaluated using the Pymol program (pymol.org).

## Results

### Clinical/Operative Analysis

A total of 28 patients with CS have been identified in our center, of which 24 patients were operated and four patients refused surgery. Sixteen patients were male (57%) and 12 were female (43%) resulting in a male to female ratio of 4:3. Consanguinity was observed in nine families (32% of families) six of which were SCS cases. The mean age of presentation was 9.3 months and the mean age at surgery was 17 months. Amongst the 24 operated patients, 22 presented earlier (mean age of 5.4 months) and had a mean age at surgery of 12.5 months. The other two patients presented later with re-synostosis at a mean age of 5.5 years. Simple CS was observed in 14 cases (50%) and complex CS in 14 cases (50 %). The most commonly encountered simple skull suture fusion was metopic suture (trigonocephaly) in six patients (21.5%) followed by unilateral coronal (anterior plagiocephaly) in four patients (14.2%) and sagittal CS (scaphocephaly), found in four patients (14.2 %).

The most common procedure performed was cranial vault reconstruction with front-orbital advancement in 20 patients (83% of surgeries) followed by posterior vault distraction in two patients (8.5%) and strip suturectomy with barrel staving strip also in two patients (8.5%). The two cases who underwent PVDO were patients who presented with increased ICP due to re-synostosis, one of which was diagnosed as Curzon syndrome and the other showed FREM1 gene which was reclassified as likely benign. Out of the 24 patients whom were operated, seven patients (29% of surgical cases) had raised ICP documented by papilledema, all of these patients were found to be syndromic cases. Table 1 demonstrates the clinical and genetic characteristics of each patient.

### Molecular Analysis

**Figure 3:**
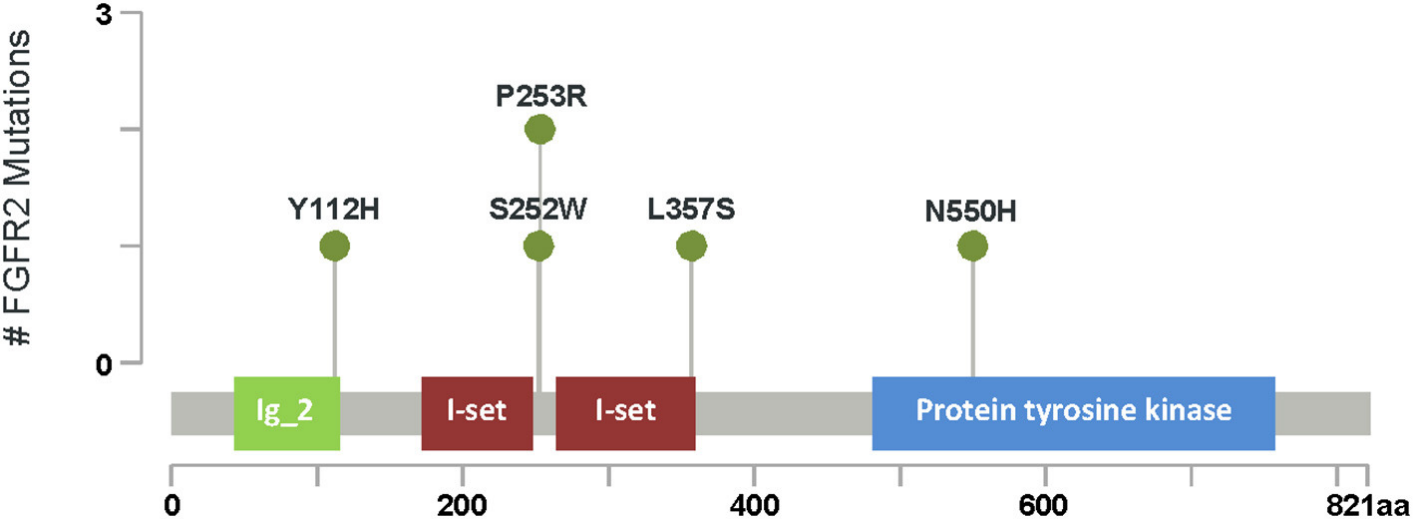
FGFR2 mutations. Lollipop plot of the major domains and protein sequence FGFR2. Green circles represent missense mutations. The length of vertical lines correlates with the frequency of respective mutation as indicated in the y-axis. ^#^ Number of *FGFR2* gene mutation seen in our cohort.

*FGFR2* gene sequencing was performed in six patients with unicoronal or bicoronal craniosynostosis, usually based on clinical and radiological findings, characteristic facial features and hand and foot findings. All six patients were found to have pathogenic *FGFR2* variants: two *de novo* and one paternally inherited variant (Figure 3).

**Figure 4:**
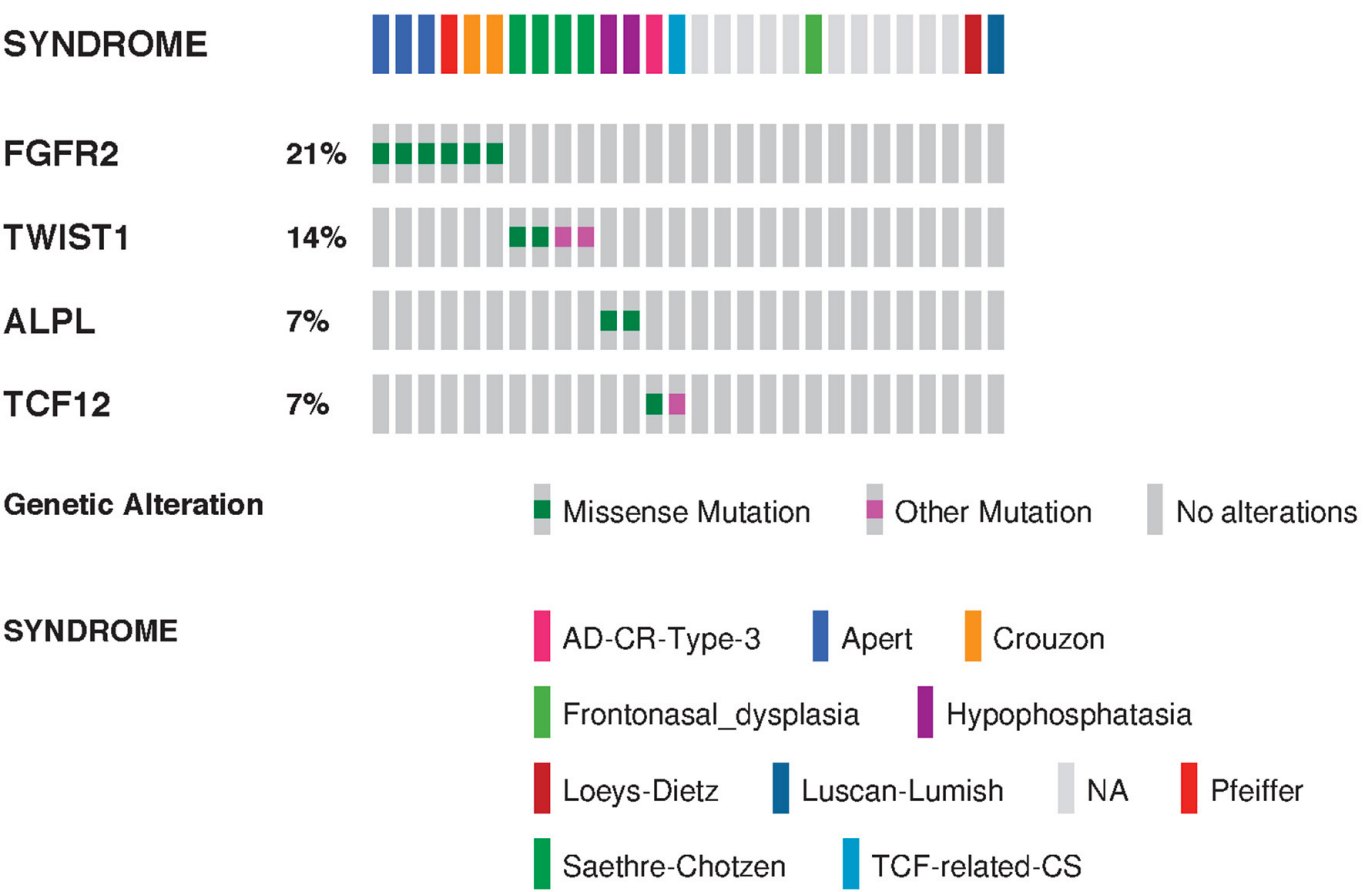
Summarized visualization of the genes mutated in craniosynostosis cohort. Data is shown for Craniosynostosis cohort of 28 patients. Columns are clustered by syndrome type where each column represent one sample. Type of genomic event and syndrome are color coded. Only variants classified as pathogenic or likely pathogenic are shown.

**Figure 5:**
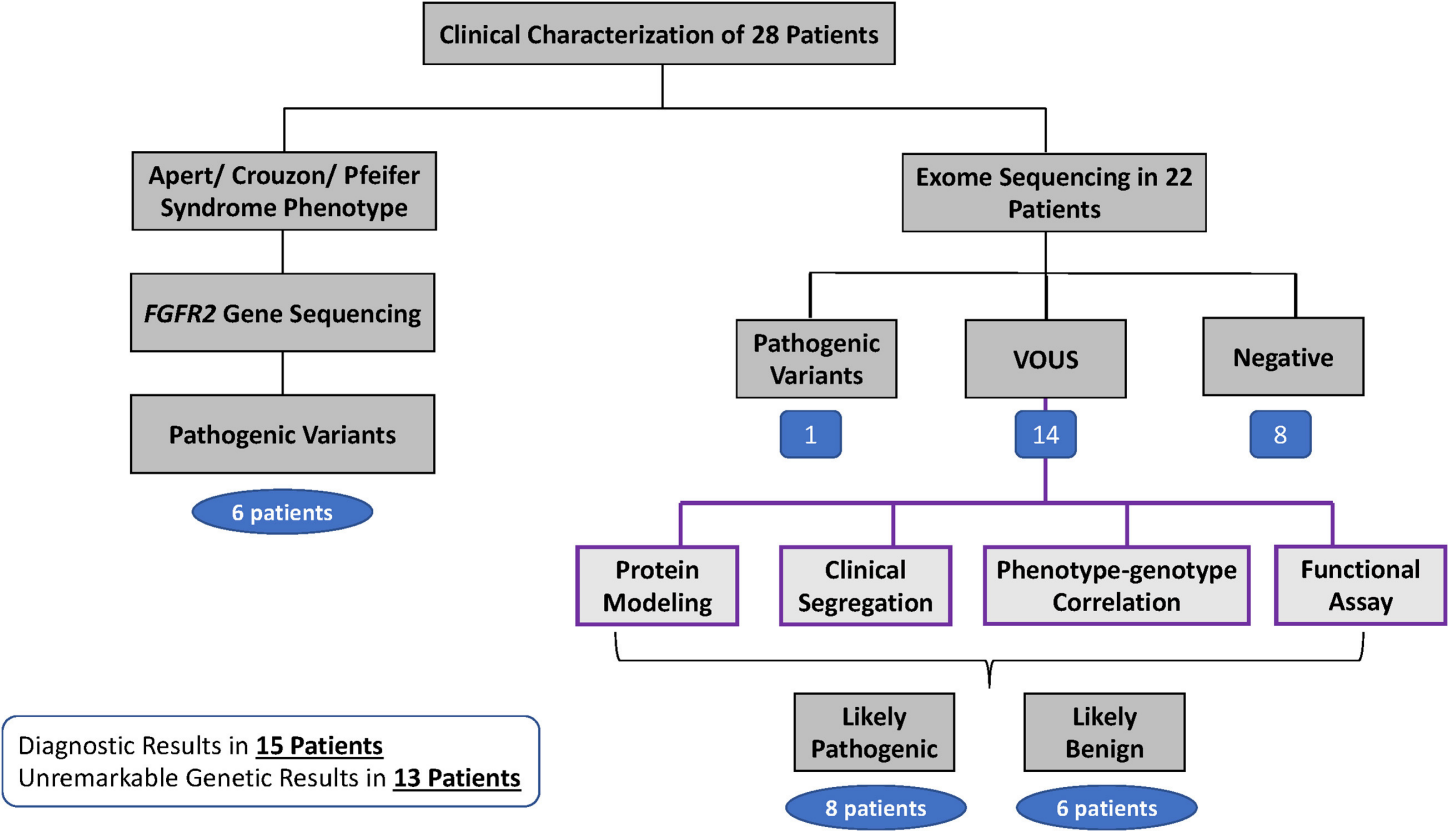
Clinical and Genetic workflow and result of 28 patients.

Whole exome sequencing (WES) was performed in 22 patients (Figure 4). Sixteen patients with isolated craniosynostosis and six patients with multiple congenital anomalies including craniosynostosis. Genetic testing identified genetic variants in 15 patients with craniosynostosis associated with other anomalies and in 10 isolated craniosynostosis patients. ES in single suture craniosynostosis yield was only 18% (2/11) as shown in Table 1. Consanguinity was found in eight families. Exome Sequencing revealed pathogenic mutations in one family (a homozygous variant in *ALPL* gene), VOUS in 14 families, and failed to detect variants that explain the phenotype in eight families (Figure 5).

#### Variants Re-classification and Protein Modeling

Fourteen novel variants were identified from exome sequencing. Re-classification was done based on clinical (phenotype-genotype) consistency, family segregation, protein structure modeling and/or functional assays. Three variants in the *TWIST1* gene were re-classified as likely pathogenic by family segregation (two paternally inherited and one maternally inherited). Two variants in the *FREM1* gene was re-classified as likely pathogenic by family segregation and one as a *de novo* variant (Table 1). Clinical consistency and family segregation suggested the pathogenicity of two variants in *TCF12* gene, and one variant in *ALPL* gene was confirmed to be likely pathogenic by clinical consistency and functional assays.

For selected novel variants, we used bioinformatics and *in silico* structural modeling to gain more insights into the molecular effects of the mutations.

The *ALPL* variant c.977G>T p.(Gly326Val) causes an amino acid change from Gly to Val at position 326. ALPL is an alkaline phosphatase that plays a central role in skeletal mineralization by controlling diphosphate levels. According to HGMD Professional 2017.3, this variant has previously been described as disease causing for hypophosphatasia (15). There is no data regarding the allele frequency in Genome Aggregation Database (gnomAD), Exome Sequencing Project (ESP), and the 1000 Genome project (1000G). Also, the variant description based on Alamut Batch (latest database available) including AlignGVD, SIFT, PolyPhen, and Mutation Taster corroborated a deleterious effect of the variant. Biochemical investigation including plasma and urine inorganic pyrophosphate (PPi) and serum PLP and urine phosphoethanolamine (PEA) are consistent with the phenotype with the [B6 (high), urinary PEA (high) and alkaline phosphatase (low)] as demonstrated in Table 1.

**Figure 6:**
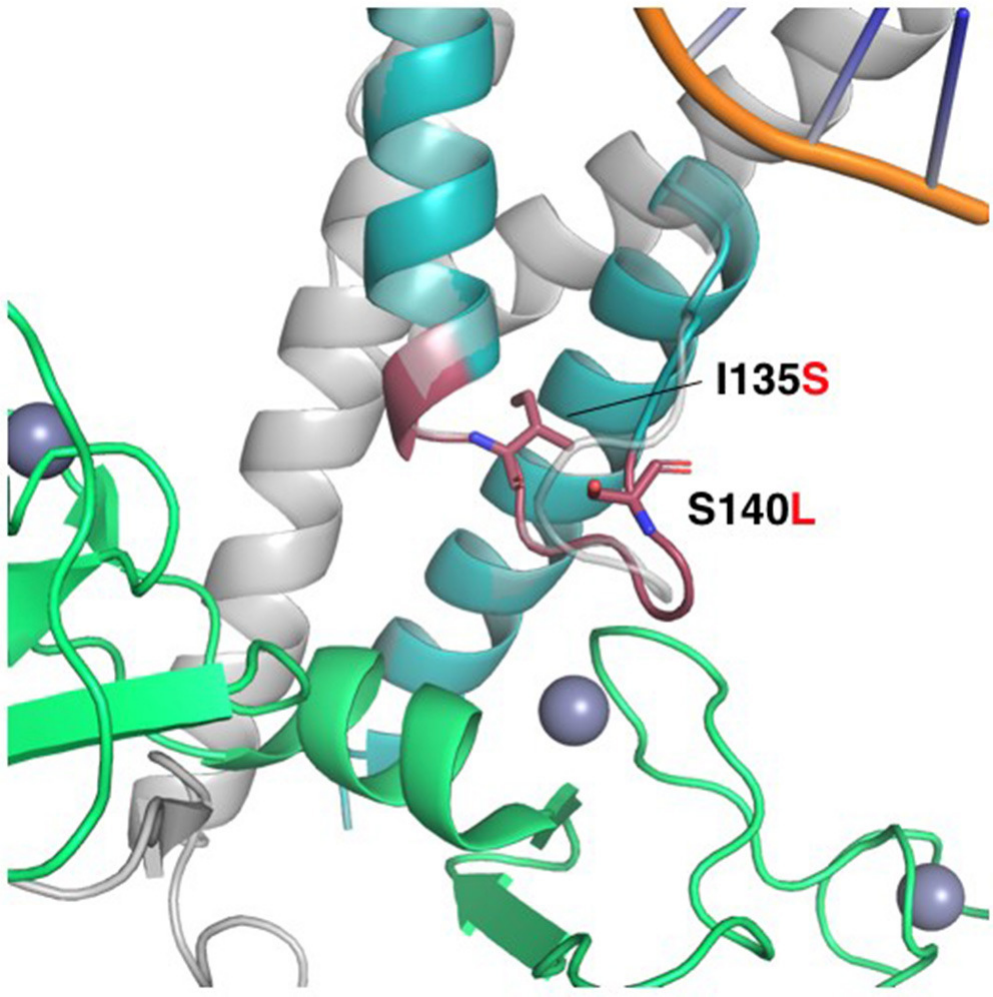
Homology model of the HLH domain from TWIST1. HLH motif (colored teal), superimposed to the SCL protein in the crystal structure of the human SCL:E47:LMO2:LDB1 complex bound to DNA (PDB ID 2YPA). LMO2 is colored green. The Lys133-Pro139 duplication is shown as a red cartoon, while the non-synonymous mutations Ile135Ser and Ser140Leu are shown as red sticks. The loop in SCL interacts with the first LIM domain of LMO2, and the duplication of this loop in TWIST1 might alter the interaction with its corresponding regulatory machinery. Ile135 is located in the interface to the second helix in the HLH, and the substitution for the polar serine might alter the orientation of the helices and compromise the stability of the complex. Ser140 is pointing to the outside of the structure, and the substitution for leucine might change the specificity of the loop to interact with other binding partners.

The twist-related protein 1 (TWIST1) is a transcriptional regulator that inhibits myogenesis. p.(Lys133_Pro139dup), p. Ile135Ser and p. Ser140Leu affect a conserved loop region connecting two alpha-helices in the basic helix-loop-helix motif (bHLH), which is essential for protein dimerization and DNA binding (PubMed: 11992718). A homology model of the bHLH domain from TWIST1 was produced by SWISS-MODEL (10), based on the crystal structure of the human SCL:E47:LMO2:LDB1 complex bound to DNA [PDB ID 2YPA (16) seq. identity = 48%, QMEAN = −0.06] (Figure 6). In the template structure, SCL forms a heterodimer with E47 through the bHLH domain while at the same time interacting with LMO2 through residues localized in the helix 2 and loop of SCL. In the TWIST1 protein, a similar complex is likely to be sustained through interactions in the bHLH domain and its loop region. Accordingly, these mutations might affect DNA binding and the capacity of TWIST1 to nucleate multi-protein complexes (Figure 6). The p.(Lys133_Pro139dup) variant has been reported in individuals affected with craniosynostosis and was observed to be *de novo* in one case (17, 18). The *TWIST1* variant c.419C>T p.(Ser140Leu) causes an amino acid change from Ser to Leu at position 140. According to HGMD professional 2019.1, a different amino acid change in the same codon, p.(Ser140Pro) (c.418T>C), has been previously reported by Ko et al. (19) in a single patient presenting a phenotype overlapping with the Saethre-Chotzen syndrome. Due to more evidence of pathogenicity based on family segregation and the protein modeling, the detected variant is classified as likely pathogenic consistent with the phenotype according to the recommendations of ACMG.

The glycines 48 and 51 are in a disordered N-terminal region of *TWIST1* that associates with p300 and KAT2B. Hence, p.(Gly48_Gly51del) might affect these interactions. However, the p.(Gly48_Gly51del) variant was detected in one of our patients (pt 21 in the Table 1) for whom familial carrier testing revealed that the variant does not segregate with the expected clinical phenotype as the healthy father carries the same variant (Figure 6, pedigree 21). Therefore, the variant is no longer considered to have potential clinical relevance and reclassified as a likely benign variant. There are no data available in gnomAD, ESP or 1000G regarding the allele frequency or variant description based on Alamut Batch (latest database available).

**Figure 7:**
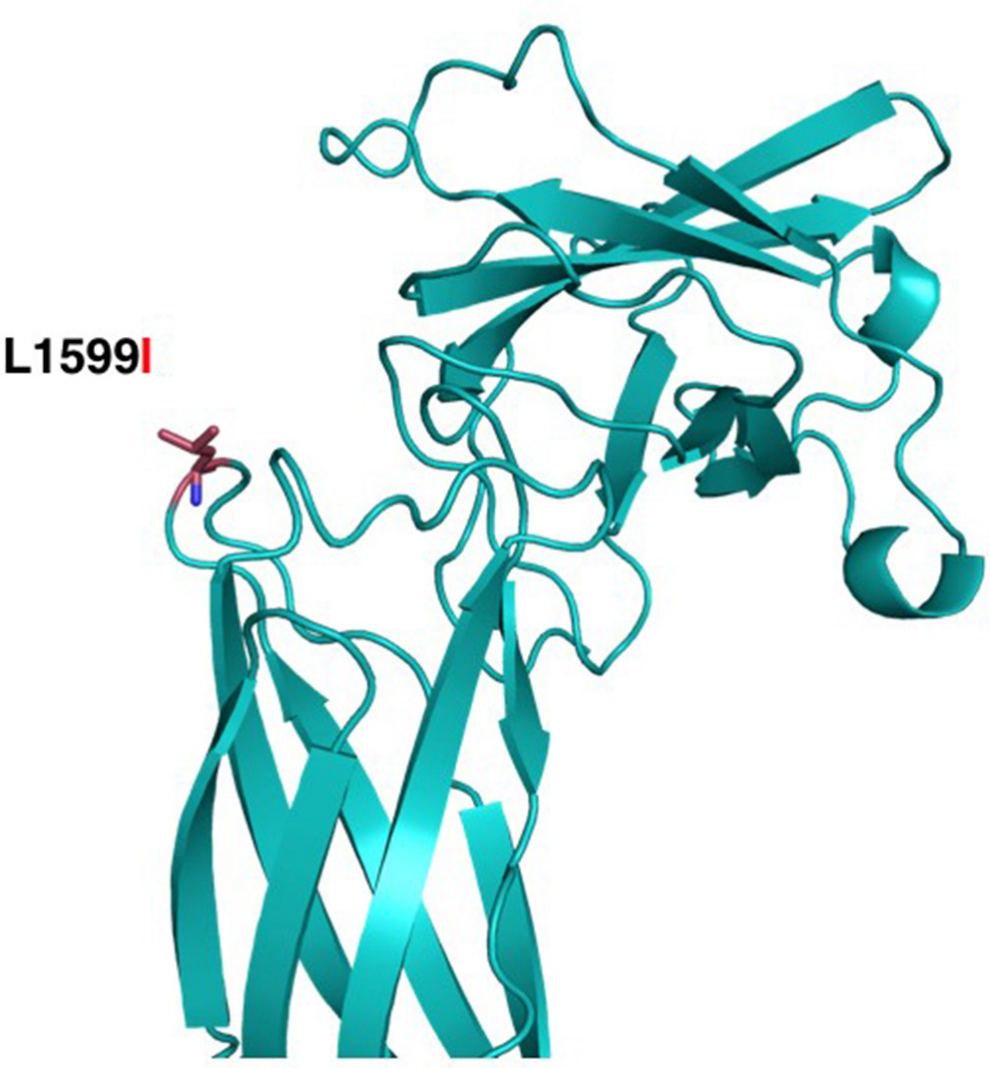
Structural modeling of Leu1599Ile in SMCHD1. The mutation is located closer to the Flexible Hinge Domain (aa 1719–1847). The substitution of Leu1599 (red sticks) for the Isoleucine is a conservative replacement pointing to the outside of the structure, which might not compromise the stability of the structure of the protein domain.

The structural maintenance of chromosomes flexible hinge domain-containing protein 1 (SMCHD1) has several functions. It is involved in epigenetic gene silencing on the female inactive X chromosome and of a subset of clustered autosomal loci in somatic cells. It has a role in DNA repair of double-strand breaks and regulates embryonic genome function. The p.Leu1599Ile mutation is in a solvent-exposed loop of a central structured domain of unknown function. trRosetta (11) as used to produce a structural model of the region containing Leu1599 (aa 1350–1850); the resulting model had an estimated TM-score of 0.447 (Figure 7). This conservative substitution does not affect the structural stability but might alter ligand binding. This variant has been observed at a frequency of <0.01%. Analysis of amino acid conservation indicates that the wild-type amino acid, Leu1599, is conserved in 11/11 primates, in 0/50 non-primate mammals, and in 0/25 non-mammalian vertebrates. The fact that amino acid conservation differs widely among species does not provide insight into the effects of amino acid substitution at this position on the structure and/or function of the protein. The physiochemical difference between Leu and Ile as measured by Grantham's distance is 5. This score is considered a “conservative” change, indicating that Leu and Ile have similar physiochemical properties (20, 21). Predictive algorithms: 0/2 deleterious; 2/2 tolerated (AGVGD, SIFT). Previously, *SMCHD1* mutations have been associated with autosomal dominant muscular dystrophy and with the Bosma Arhinia and Microphthalmia Syndrome (BAMS), a rare condition characterized by eye and nose abnormalities. Due to lack of pathological evidence and clinical consistency, this variant is considered as a likely benign variant.

**Figure 8:**
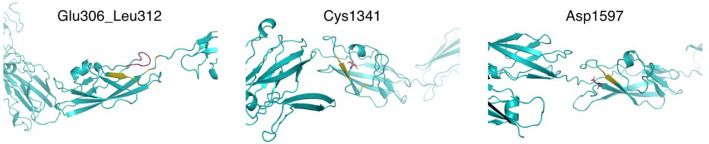
Structural modeling of FREM1 mutations. Models of the different mutation-containing regions in FREM1 were produced by trRosetta. Left: region showing Glu306_Leu312 in CSPG1 and nearby residues (estimated TM-score = 0.546). The segment Glu306_Leu312 is colored red and the predicted contacts are shown in olive. Center: region containing CSPG9, highlighting Cys1341 in red sticks and predicted contacts in olive (estimated TM-score = 0.547). Right: region containing CSPG11, highlighting Asp1597 in red sticks and predicted contacts in olive (estimated TM-score = 0.565).

The Fras-related extracellular matrix protein 1 (FREM1) contributes to cranio-facial morphogenesis, renal morphogenesis and multicellular organism development. The protein has an N-terminal signal peptide and contains 12 cadherin-like CSPG (CSPG1-12) repeats followed by a Calx-beta and C-type Lectin domains (Figure 8). p. (Glu306_Leu312dup) dup alters a loop segment of the CSPG1 (spanning 296–390), according to RaptorX and trRosetta predictions (11, 22). P.Cys1341Trp is predicted to cause significant steric clashes in the CSPG9 domain by targeting the buried Cys1341. Asp1597 is flanking CSPG11 and possibly interacts with calcium ions. Hence, the variant p.Asp1597Tyr might affect calcium binding. CSPG elements can interact with growth factors and mediate interaction with Fras1 and Frem2. Mutations in these regions possibly contribute to developmental defects. Furthermore, based on the clinical information provided, previously performed analyses for the family members, the information available for the variants and the features described for *FREM1*-related trigonocephaly type 2, we conclude that the variants c.4023C>G p.(Cys1341Trp) and c.4789G>T p.(Asp1597Tyr) are in cis and likely segregate with the craniosynostosis phenotype in this family. However, given that the sibling that is heterozygous for the other variant, c.4564G>A p.(Val1522Met), might also be affected with frontal and/or back bossing and that incomplete penetrance has been reported for the related disorder, it cannot be excluded that the variant c.4564G >A has a clinical effect and therefore it remains classified as variant of uncertain significance.

*TCOF1* encodes for the serine/alanine-rich protein Treacle. This nucleolar protein acts as a regulator of RNA polymerase I by connecting it with enzymes responsible for ribosomal processing modification. Treacle is a mostly unstructured protein containing highly polar repeat motif regions, the Treacle domains. The mutation p.Ser875_Glu880del affects the disordered fourth Treacle domain, and deletes four positive charges and two serines, one of which is a phosphorylation site (Ser875). Previously, *TCOF1* variants with premature-termination codons were associated with an autosomal dominant craniofacial development disorder, the Treacher Collins Syndrome (23).

The mostly disordered transcription factor 12 (TCF12) contains a basic helix-loop-helix domain, two activation domains and a Rep domain. The mutation p.Pro214Arg affects a proline-rich region of unknown function. It disrupts a putative site for SH3 domain binding sites and MAP kinase phosphorylation. Several *TCF12* mutations have been identified in *TCF12* from individuals with craniosynostosis (24, 25).

Six variants were re-classified as likely benign by family segregation and clinical consistency, respectively. Variants in *ALX4* (pt 16 in the Table 1), *SMCHD1* and *TCOF1* (pt 19 in the Table 1), *TWIST1* gene (pt 21 in the Table 1), *TGFBR2* (pt 26 in the pedigree), and *SETD2* (pt 27 in the Table 1) were re-classified as likely benign based on clinical consistency and family segregation (Table 1).

## Discussion

In Western countries, the CS incidence has been reported to affect from 1 in 2,000–2,500 live births (3) to as many as 1 in 1,400 live births (26), with the most common types being sagittal synostosis (40–55%) and metopic synostosis. CS occurs pre-dominantly in boys, with a male-to-female ratio of 4:1 (27). Incidence in the Netherlands in a multicenter study showed an incidence of metopic synostosis and sagittal synostosis of 1.9 and 2.8 per 10,000 live births in the, respectively, with significant increase in the incidence and the proportion of metopic synostosis over the study period, suggesting that metopic synostosis is on the rise (28). In Germany, prevalence of is 4.8/10,000 births with a male: female ratio of 4.7:1 with sagittal suture synostosis being the most common (29). In the middle east, 116 cases were studied in Syria. Out of these patients, the male to female ratio was found to be 1.1:1. The corresponding disease type distribution was scaphocephaly 22%, occicephaly 15%, trigonocephaly 24%, plagiocephaly 17%, brachycephaly 10%, cloverleaf 5%, unclassified 5%, making sagittal CS the top deformity. The ratio of syndromic to non-syndromic cases was 19–81%, respectively (30). In Saudi Arabia, to our knowledge, there are no studies of the incidence of CS nationwide. However, Aziza et al. (31) reported that 33% of all patients presenting to their center with craniofacial (CF) anomalies presented with CS. A total of 43.6% were reported to be syndromic with Apert, Crouzon, and Seather-Chotzen as most common syndromes. A family history of CF and other anomalies was observed more in children born to parents of consanguineous marriages than in those whose parents were unrelated, comprising the effect of consanguinity on familial inheritance of these anomalies (31). Crouzon syndrome, Treacher-Collins syndrome, Angelman syndrome, and Turner syndrome were reported to have a prevalence of 0.2 per 10,000 children each in another center nationally (32). In regards to age at presentation, various reports investigated the usual age of presentation of craniosynostosis patients and showed a variability between an age of 8.9 months and 2.01 (± 2.57 years) (33, 34) which is consistent with the mean age at presentation of 9.3 months in our study.

Our study represents a single referral center experience. In our population we found that the mean age of presentation was 9.3 months. Our patients are referred to us from within our center and many other locations, which may explain delay in presentation. From our population of 28 patients, we observed a male: female ratio of 4:3. We encountered an equal number of SCS cases (14 cases) and NSCS cases (14 cases). Though NSCS was more common than syndromic, this may be explained by the fact that families detect syndromic children quicker and seek medical help earlier, leaving out some children with simple suture fusion who go undetected and unmanaged. The most commonly encountered simple skull suture fusion was metopic suture (trigonocephaly) in six patients (21.5%) followed by unilateral coronal (plagiocephaly) in four patients (14.2%) and sagittal CS (scaphocephaly), found in four patients (14.2%). Again, despite sagittal suture synostosis being the most common suture fusion world-wide, this may be also explained by the fact that metopic is noticed by families who seek help earlier. This could be an indication for the need for increased family education and public health on such conditions in the country. Two thirds of the children who had consanguineous parents were found to be syndromic, confirming that consanguinity does play a clear role in CS incidence.

Surgical treatment of CS depends on the age of the patient, ICP, type of deformity and its severity. Options include suturectomy, springs, FOBA+ CR, posterior vault reshaping, and PVDO (1). For NSCS, options, such as suturectomy or springs are used at earlier ages. Their indications are specific, and springs requires a second surgery for removal. FOB+ CR or PVDO are used in patients presenting at ages older than 6 months. These options give better skull expansion and relieve increased intracranial pressure faster. Iida et al. (35) proposed a new strategy, which involves performing FOBA first, followed by PVDO for severe SCS and report more favorable results in those patients.

In our population, the most common procedure performed was FOBA+CR for most of our patients as we had no patients with lambdoid synostosis. The second most commonly performed procedures were suturectomy and barrel staving for sagittal synostosis in children <4 months old, and PVDO. PVDO was performed in older children with a mean age of who presented with resynostosis after primary surgery at due age. PVDO was used for two cases as they were both SCS patients who presented with increased ICP one of which was diagnosed as Curzon syndrome and the other showed *FREM1* gene which was reclassified as likely benign with a total mean age at surgery of 17 months. Amongst the 24 operated patients, 22 presented earlier with a mean age of 5.4 months and a mean age at surgery of 12.5 months. The other two patients presented later with re-synostosis at mean age of 5.5 years.

Jabs et al. (36), were the first to identify a genetic mutation in relation to CS. They described a mutation in the *MSX2* gene in a patient with Boston type craniosynostosis (37). Since then, about 57 genes mutations have been identified to be linked to CS (37). Molecular and genetic testing has advanced hugely in the recent years. This had led to aiding the identification of both SCS and NSCS. However, variable phenotype expression of genetic aberration in the same mutation can lead to different disorders with different expression, due to changes in gene penetrance and regulatory mechanisms (2). Genetic testing is not limited to SCS. Several authors have proven clear links between NSCS and genetic defects. Sewda et al. (5) identified five novel, heterozygous coding single nucleotide variants (SNV) predicted to be pathogenic. These variants involve the following genes: *ALX4, BBS9, EFNB1*, and *TWIST1* (3). They also identified 18 previously identified *SNV* (3). The most commonly diagnosed SCS are Apert, Crouzon, Pfeiffer, Muenke, and Saethre-Chotzen syndromes. All of these are inherited as autosomal dominant (AD) conditions (1). Single genes have been implicated in the above-mentioned syndromes. These are fibroblast growth factor receptor 2 *(FGFR2), FGFR3, TWIST1, ERF, TCF12*, and *EFNB1* (32). Apart from these single-gene mutations, the second group is caused by chromosomal rearrangements (~13%) (38).

To our knowledge, there are no genetic studies of craniosynostosis in Saudi Arabia. Aziza et al. (31) and Al Salloum et al. (32) reported SCS, such as Apert, Crouzon syndrome, Saethre-Chotzen syndrome, however, genetic correlation for the syndromes were not identified and/or studied. In our study, a genetic diagnosis has been identified for 15 out of 28 patients as follows: Hypo-phosphasia (two patients), Apert syndrome (three patients), Crouzon syndrome (two patients), Seather-Chotzen, (three patients), *TCF12* related CS (one patient), Pfeiffer syndrome Type 1 (one patient) *FREM1*-related trigonocephaly type 2 (two patient) and AD CR type 3 (one patient). Thus, Apert syndrome and Seather-Chotzen were the most common amongst our population as (Figure 3). According to our study, almost all of CR genes (except one) were dominant, while two variants were *de novo* in the *FGFR2* gene. While we revealed many novel VOUS variants (14 out of 22), the advantage of phenotype-genotype consistency in our population made it possible to reclassify those variants, in which we were able to re-classify 50% of them to be likely pathogenic.

## Conclusion

CS is a complex craniofacial deformity. Our study is a retrospective chart review in a single referral center cohort. To our knowledge this is the first study in Saudi Arabia correlating clinical and genetic characteristics of CS patients. In our population, metopic suture fusion was observed to be the most commonly affected suture. The incidence of SCS cases was found to be slightly higher in our population probably due to easier recognition of the deformity. Direct *FGFR2* sequencing showed two pathogenic *de novo* variants and four inherited variants, while exome sequencing showed one homozygous variant in *ALPL* gene. Novel pathogenic heterozygous variants were revealed in *TWIST1, ALPL, TCF12*, and *FREM1* genes that were confirmed by phenotype-genotype consistency, family segregation, protein modeling and/or functional assays, presenting Seather-Chotzen and Apert syndromes to be the most common in the Saudi population. Further studies are needed to investigate the characteristics of CS in our population in a multicenter setting.

## Data Availability Statement

The datasets presented in this study can be found in online repositories. The names of the repository/repositories and accession number(s) can be found at: https://databases.lovd.nl/shared/individuals/00334866.

## Ethics Statement

The studies involving human participants were reviewed and approved by The Institutional Review Board in King Saud University. Written informed consent to participate in this study was provided by the participants' legal guardian/next of kin. Written informed consent was obtained from the minor(s)' legal guardian/next of kin for the publication of any potentially identifiable images or data included in this article.

## Author Contributions

TA and MA developed the theory, encouraged the others to investigate further these patients, wrote and edited the final manuscript, and drafted the figures. OB and WA collected the clinical, biochemical and radiological data, reviewed the literature, and also helped in drafting the manuscript. NA and HA worked on the Laboratory DNA extraction, sanger sequencing to verify the WES findings, and to complete the family segregation. IA neurosurgeon and TA are the plastic surgeons who did the surgical procedure and commented on the radiological findings. AB and MB verified the analytical methods and supervised the laboratory findings of this work. All authors discussed the results and contributed to the final manuscript, provided critical feedback, and helped shape the research, analysis and manuscript.

## Conflict of Interest

The authors declare that the research was conducted in the absence of any commercial or financial relationships that could be construed as a potential conflict of interest.
