# Supplementary material for: Clinical and Genetic Characterization of Craniosynostosis in Saudi Arabia
Source: Front Pediatr. 2021 Apr 16;9:582816. doi: 10.3389/fped.2021.582816 (PMC8085561; doi:10.3389/fped.2021.582816)
Supplement: Supplementary file 1 [file Data_Sheet_1.DOCX]

## **Supplementary Material:**

## **Structural and bioinformatic evaluation of selected variants.**

**Protein:** TWIST1; Twist-related protein 1

**Function:** Transcriptional regulator. Inhibits myogenesis by sequestrating E proteins, inhibiting trans-activation by MEF2, and inhibiting DNA-binding by MYOD1 through physical interaction. Activates transcription as a heterodimer with E proteins and regulates gene expression differentially depending on the dimer composition.

**Mutations:** p.(Gly48_Gly51del), p.(Lys133_Pro139dup), p.Ile135Ser, p.Ser140Leu

**Structural analysis:** The protein has a hydrophilic N terminus, followed by a repeat region that encodes a glycine rich sequence and a conserved basic helix-loop-helix motif in the C-terminal half (Deng, Pan et al. 2015).

**Predicted effect:** Gly48_Gly51 are located in a region predicted to be disordered (Wang, Li et al. 2016), however the N-terminus of TWIST is known to be an interacting site for both p300 and KAT2B proteins, inhibiting histone acetyltransferase activity and p300-dependent transcription (Hamamori, Sartorelli et al. 1999). The deletion of this glycine-rich stretch might affect the proper interaction of this region with its partner proteins.

Lys133_Pro139 are highly conserved residues (Ashkenazy, Abadi et al. 2016) located in the loop connecting the two alpha-helices in the helix-loop-helix motif (Figure S1). A homology model was produced by SWISS-MODEL (Waterhouse, Bertoni et al. 2018) for this region, based on the crystal structure of the human SCL:E47:LMO2:LDB1 complex bound to DNA (PDB ID 2YPA (El Omari, Hoosdally et al. 2013)). TWIST1 has a sequence identity of 48% to the SCL protein, and the resulting model had a QMEAN value of -0.06. In the template structure, the SCL:LMO2 interaction occurs mainly through residues localized in the helix 2 and loop of SCL and the first LIM domain of LMO2. These interactions help the SCL:E47 heterodimer nucleate a “core” multiprotein complex by binding to the adaptor protein LMO2 and its interacting partner. This complex then acquires further specificity through recruitment of additional protein partners. In the TWIST1 protein, a similar complex is likely to be sustained through interactions in the loop region. The duplication of the residues Lys133_Pro139 in this region is likely to alter such interactions, thus having a negative effect on its regulatory functions.

Ile135 is located in the same loop region, at the end of the first helix in the HLH motif (Figure S1). Furthermore, it is located at the interface between helices 1 and 2, and its substitution by the polar serine is bound to alter the orientation of the helices with respect to each other and to compromise the stability of the regulatory complex.

Ser140 is also located in the loop connecting the two alpha helices. The loop extends to the outside of the complex in the template structure, and this position could potentially contribute to other interactions with binding partners. The change for the larger and non-polar leucine might affect the specificity of such interactions.

Mutations affecting the helix 1 and loop regions in TWIST1 have been identified previously in patients with Saethre-Chotzen syndrome (Howard, Paznekas et al. 1997). These include Ile135ins7 (AALRKII) and Pro139ins7 (KIIPTLP), which highlights the importance of this loop for the proper function of TWIST1.

**
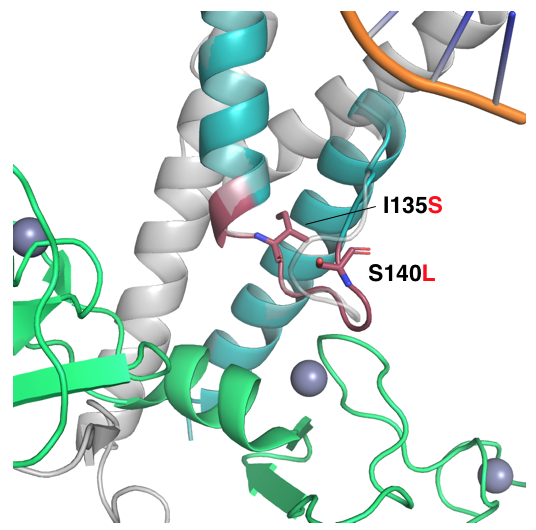
Figure S1. Homology model of the HLH motif from TWIST1**

HLH motif (colored teal), superimposed to the SCL protein in the crystal structure of the human SCL:E47:LMO2:LDB1 complex bound to DNA (PDB ID 2YPA). LMO2 is colored green. The Lys133-Pro139 duplication is shown as a red cartoon, while the non-synonymous mutations Ile135Ser and Ser140Leu are shown as red sticks. The loop in SCL interacts with the first LIM domain of LMO2, and the duplication of this loop in TWIST1 might alter the interaction with its corresponding regulatory machinery. Ile135 is located in the interface to the second helix in the HLH, and the substitution for the polar serine might alter the orientation of the helices and compromise the stability of the complex. Ser140 is pointing to the outside of the structure, and the substitution for leucine might change the specificity of the loop to interact with other binding partners.

**References**

Ashkenazy, H., S. Abadi, E. Martz, O. Chay, I. Mayrose, T. Pupko and N. Ben-Tal (2016). "ConSurf 2016: an improved methodology to estimate and visualize evolutionary conservation in macromolecules." Nucleic Acids Research **44**(W1): W344-W350.

Deng, X., H. Pan, J. Wang, B. Wang, Z. Cheng, L. Cheng, L. Zhao, H. Li and X. Ma (2015). "Functional Analysis of Two Novel Mutations in TWIST1 Protein Motifs Found in Ventricular Septal Defect Patients." Pediatr Cardiol **36**(8): 1602-1609.

El Omari, K., S. J. Hoosdally, K. Tuladhar, D. Karia, E. Hall-Ponsele, O. Platonova, P. Vyas, R. Patient, C. Porcher and E. J. Mancini (2013). "Structural basis for LMO2-driven recruitment of the SCL:E47bHLH heterodimer to hematopoietic-specific transcriptional targets." Cell Rep **4**(1): 135-147.

Hamamori, Y., V. Sartorelli, V. Ogryzko, P. L. Puri, H. Y. Wu, J. Y. Wang, Y. Nakatani and L. Kedes (1999). "Regulation of histone acetyltransferases p300 and PCAF by the bHLH protein twist and adenoviral oncoprotein E1A." Cell **96**(3): 405-413.

Howard, T. D., W. A. Paznekas, E. D. Green, L. C. Chiang, N. Ma, R. I. Ortiz de Luna, C. Garcia Delgado, M. Gonzalez-Ramos, A. D. Kline and E. W. Jabs (1997). "Mutations in TWIST, a basic helix-loop-helix transcription factor, in Saethre-Chotzen syndrome." Nat Genet **15**(1): 36-41.

Wang, S., W. Li, S. Liu and J. Xu (2016). "RaptorX-Property: a web server for protein structure property prediction." Nucleic Acids Research **44**(W1): W430-W435.

Waterhouse, A., M. Bertoni, S. Bienert, G. Studer, G. Tauriello, R. Gumienny, F. T. Heer, T. A. P. de Beer, C. Rempfer, L. Bordoli, R. Lepore and T. Schwede (2018). "SWISS-MODEL: homology modelling of protein structures and complexes." Nucleic Acids Res **46**(W1): W296-w303.

**Protein:** SMCHD1; Structural maintenance of chromosomes flexible hinge domain-containing protein 1

**Function:** Structural maintenance of chromosomes flexible hinge domain-containing protein 1 *(SMCHD1)* is a non-canonical member of the structural maintenance of chromosomes (SMC) protein family (UniProt 2019). SMCHD1 is involved in epigenetic gene silencing and chromosome organization on the female inactive X chromosome by promoting the spreading of heterochromatin. It is also required for silencing of a subset of clustered autosomal loci in somatic cells. Additionally, it has been demonstrated that SMCHD1 also has a role in DNA repair of double-strand breaks (Coker and Brockdorff 2014). More recently, has emerged as a critical regulator of embryonic genome function (Schall, Ruebel et al. 2019).

**Mutation:** p.Leu1599Ile

**Structural analysis:** SMCHD1 is a 230 kDa protein grouped in the SMC family of chromosomal proteins based on the presence of an SMC hinge domain (Blewitt, Gendrel et al. 2008). However, SMCHD1 is a non-canonical family member and shares limited similarity with other SMC proteins, differing in ATPase domain structure and its inability to form a stable multimeric complex (Coker and Brockdorff 2014). SMCHD1 contains a GHKL (gyrases, Hsp90, histidine kinase, and MutL) domain containing an N-terminal region (Iyer, Abhiman et al. 2008), as well as a large central domain, and a C‐terminal Hinge Domain, which is responsible for forming the DNA interacting dimer. It is also possible that this is an essential determinant of the specificity of the DNA-protein interaction (Hirano and Hirano 2002).

**Predicted effect**: p.Leu1599Ile mutation is located in the long central domain between the GHKL and the Flexible Hinge Domains in a predicted structured region (Wang, Li et al. 2016) of unknown function. trRosetta (Yang, Anishchenko et al. 2020) was used to produce a structural model of the region containing Leu1599 (aa 1350-1850); the resulting model had an estimated TM-score = 0.447 (Figure S2).

Heterozygous germline mutations in the SMCHD1 gene have been identified in type 2 Facio-Scapulo-Humeral muscular dystrophy (FSHD2), an autosomal dominant muscular dystrophy. More recently, germline SMCHD1 mutations have been found in patients affected with Bosma Arhinia and Microphthalmia Syndrome (BAMS), an extremely rare condition characterized by the absence of the nose with or without ocular defects (Dion, Roche et al. 2019). However, potentially deleterious variants have been described mainly in the N-terminal region and the Hinge domain but not in the central domain.

**
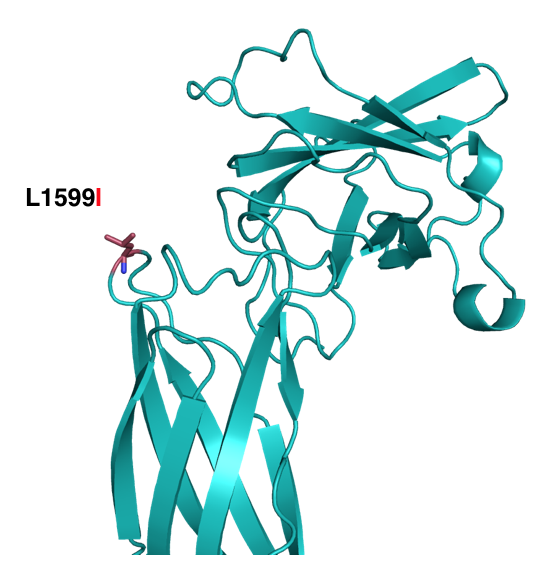
**

**Figure S2. Model of the region surrounding the mutation p.Leu1599Ile in SMCHD1**.

The mutation is located closer to the Flexible Hinge Domain (aa 1719-1847). The substitution of Leu1599 (red sticks) for the Isoleucine is a conservative replacement pointing to the outside of the structure, which might not compromise the stability of the structure of the protein domain.

**References**

Blewitt, M. E., A. V. Gendrel, Z. Pang, D. B. Sparrow, N. Whitelaw, J. M. Craig, A. Apedaile, D. J. Hilton, S. L. Dunwoodie, N. Brockdorff, G. F. Kay and E. Whitelaw (2008). "SmcHD1, containing a structural-maintenance-of-chromosomes hinge domain, has a critical role in X inactivation." Nat Genet **40**(5): 663-669.

Coker, H. and N. Brockdorff (2014). "SMCHD1 accumulates at DNA damage sites and facilitates the repair of DNA double-strand breaks." J Cell Sci **127**(Pt 9): 1869-1874.

Dion, C., S. Roche, C. Laberthonniere, N. Broucqsault, V. Mariot, S. Xue, A. D. Gurzau, A. Nowak, C. T. Gordon, M. C. Gaillard, C. El-Yazidi, M. Thomas, A. Schlupp-Robaglia, C. Missirian, V. Malan, L. Ratbi, A. Sefiani, B. Wollnik, B. Binetruy, E. Salort Campana, S. Attarian, R. Bernard, K. Nguyen, J. Amiel, J. Dumonceaux, J. M. Murphy, J. Dejardin, M. E. Blewitt, B. Reversade, J. D. Robin and F. Magdinier (2019). "SMCHD1 is involved in de novo methylation of the DUX4-encoding D4Z4 macrosatellite." Nucleic Acids Res **47**(6): 2822-2839.

Hirano, M. and T. Hirano (2002). "Hinge-mediated dimerization of SMC protein is essential for its dynamic interaction with DNA." EMBO J **21**(21): 5733-5744.

Iyer, L. M., S. Abhiman and L. Aravind (2008). "MutL homologs in restriction-modification systems and the origin of eukaryotic MORC ATPases." Biol Direct **3**: 8.

Schall, P. Z., M. L. Ruebel and K. E. Latham (2019). "A New Role for SMCHD1 in Life's Master Switch and Beyond." Trends Genet **35**(12): 948-955.

UniProt, C. (2019). "UniProt: a worldwide hub of protein knowledge." Nucleic Acids Res **47**(D1): D506-D515.

Wang, S., W. Li, S. Liu and J. Xu (2016). "RaptorX-Property: a web server for protein structure property prediction." Nucleic Acids Research **44**(W1): W430-W435.

Yang, J., I. Anishchenko, H. Park, Z. Peng, S. Ovchinnikov and D. Baker (2020). "Improved protein structure prediction using predicted interresidue orientations." Proc Natl Acad Sci U S A **117**(3): 1496-1503.

**Protein**: FREM1; Fras-related extracellular matrix protein 1

**Function:** FREM1 is an extracellular matrix protein that interacts with FREM2 and FRAS1 during embryonic development. It is involved in protein as well as carbohydrate binding, cell-matrix adhesion and cell communication. It is reported to involve in cranio-facial morphogenesis, renal morphogenesis and multicellular organism development.

**Mutations:** p.(Glu306_Leu312dup), p.Cys1341Trp, p.Asp1597Tyr

**Structural analysis:** The protein has a N-terminal signal peptide and contains 12 cadherin-like CSPG (CSPG1-12) repeats followed by a Calx-beta and C-type Lectin domains. The protein is highly ordered (>98%) and composed mainly of β-sheets and coils.

**Predicted effect:** Comparing RaptorX (Wang, Li et al. 2016) and trRosetta (Yang, Anishchenko et al. 2020) predictions for Frem1, Glu306_Leu312 is mainly a loop segment (Figure S3 Left). Importantly, it is a part of CSPG1 (spanning 296-390).

Cys1341Trp is located in CSPG9 but lack evidence for Cys1341 in disulfide bond formation. As per ConSurf (Ashkenazy, Abadi et al. 2016) predictions, Cys1341 is a buried, structurally important and conserved residue (Figure S3 Center).

Asp1597 is flanking CSPG11 (Figure S3 Right). The aspartic acid possibly interacts with calcium ions (Smyth, Du et al. 2004). Hence Asp1597Tyr substitution mutation seems to affect calcium ion interaction due to charge neutralization. This is supported by the prediction that ASP1597 is an exposed, functionally important and highly conserved residue (Ashkenazy, Abadi et al. 2016).

The lack of homology-based models limits the interpretation of mutation effects on Frem1 structure and functions. However, studies suggest that the CSPG elements can interact with growth factors and mediate interaction with Fras1 and Frem2 (Smyth, Du et al. 2004). Hence structurally or functionally important residue mutations in these regions possibly contribute to developmental defects.

**
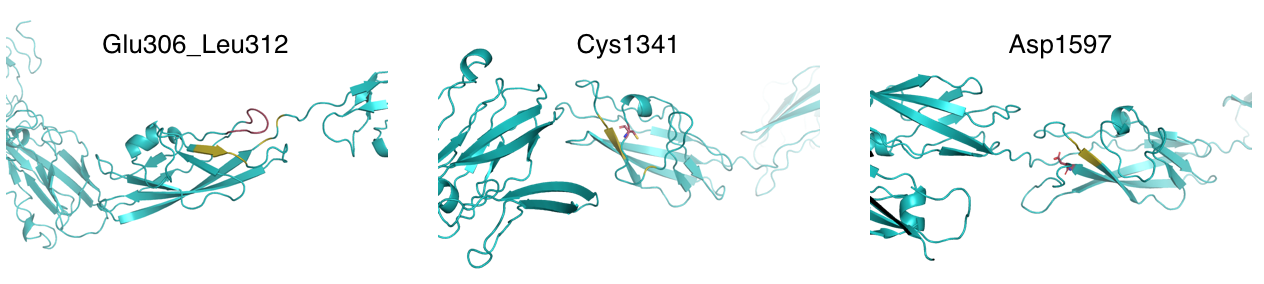
**

**Figure S3. De novo protein model of FREM1 produced by trRosetta**

**Left**: Model segment (estimated TM-score=0.546) showing Glu306_Leu312 and nearby residues. The segment Glu306_Leu312 is colored red and the predicted contacts are shown in olive. **Center**: Model segment (estimated TM-score=0.547) highlights Cys1341 is red and predicted contacts in olive. **Right**: Model segment (estimated TM-score=0.565) highlights Asp1597 is in red and predicted contacts in olive.

**References**

Ashkenazy, H., S. Abadi, E. Martz, O. Chay, I. Mayrose, T. Pupko and N. Ben-Tal (2016). "ConSurf 2016: an improved methodology to estimate and visualize evolutionary conservation in macromolecules." Nucleic Acids Res **44**(W1): W344-350.

Smyth, I., X. Du, M. S. Taylor, M. J. Justice, B. Beutler and I. J. Jackson (2004). "The extracellular matrix gene Frem1 is essential for the normal adhesion of the embryonic epidermis." Proc Natl Acad Sci U S A **101**(37): 13560-13565.

Wang, S., W. Li, S. Liu and J. Xu (2016). "RaptorX-Property: a web server for protein structure property prediction." Nucleic Acids Research **44**(W1): W430-W435.

Yang, J., I. Anishchenko, H. Park, Z. Peng, S. Ovchinnikov and D. Baker (2020). "Improved protein structure prediction using predicted interresidue orientations." Proc Natl Acad Sci U S A **117**(3): 1496-1503.

**Protein:** TCOF1: Treacle protein

**Function:** TCOF1 is a gene that encodes for the serine/alanine-rich protein Treacle. This nucleolar protein acts as a regulator of RNA polymerase I by connecting RNA polymerase I with enzymes responsible for ribosomal processing modification. The protein is especially crucial for neural crest specification: following the monoubiquitination by the BCR (KBTBD8) complex associates with NOLC1 and acts as a platform to connect RNA polymerase I with enzymes responsible for ribosomal processing and modification, leading to remodeling the translational program of differentiating cells in favor of neural crest specification (UniProt 2019).

**Mutation:** p.Ser875_Glu880del

**Structural analysis:** Treacle is a relatively simple 144 kDa protein that consists of at least three: distinct domains, including unique amino and carboxy termini and a characteristic central repeat domain (Dixon, Edwards et al. 1997, Wise, Chiang et al. 1997). Putative nuclear export and import signals are seen at the N-terminus and C-terminus, respectively. It has been reported that the C-terminal domain is essential for the nucleolar localization of Treacle (Marsh, Dixon et al. 1998)and that perhaps the intracellular localization of Treacle is very dynamic. Within the central domain, Treacle contains multiple casein kinase II and protein kinase C phosphorylation site repeats. (Sakai and Trainor 2009).

**Predicted effect**: The mutation p.Ser875_Glu880del is located in a region predicted to be disordered by RaptorX (Wang, Li et al. 2016), at the fourth Treacle domain of the protein, according to PFAM (El-Gebali, Mistry et al. 2019).

There is not enough information to provide a reliable model or hypothesis of the mutation's effects on protein structure/function. However, mutations in the TCOF1 gene have been associated with Treacher Collins syndrome (TCS), a rare congenital birth disorder characterized by severe craniofacial defects. Many mutations responsible for Treacher Collins syndrome are localized in the hot spots in exons 10, 13, 15, 16, 23, and 24 (Splendore, Jabs et al. 2003). The most commonly occurring mutations of the TCOF1 gene include deletions, which cause a shift of the reading frame, formation of the termination codon, and shortening of the protein product.

Studies have been reported mutations in the region near to the mutation c.2623_2640del p.Ser875_Glu880del. For instance, the c.2626_2627del in exon 15 that encodes repetitive motifs was identified in a TCS patient and caused the formation of a stop codon two amino acids later resulting in a truncated protein (Conte, D'Apice et al. 2011). Also, a de *novo* mutation was identified at c.2629G>A p.Val877Met, which does not alter amino acids conserved in known orthologs of TCOF1 (Teber, Gillessen-Kaesbach et al. 2004).

**References**

Conte, C., M. R. D'Apice, F. Rinaldi, S. Gambardella, F. Sangiuolo and G. Novelli (2011). "Novel mutations of TCOF1 gene in European patients with Treacher Collins syndrome." BMC Med Genet **12**: 125.

Dixon, J., S. J. Edwards, I. Anderson, A. Brass, P. J. Scambler and M. J. Dixon (1997). "Identification of the complete coding sequence and genomic organization of the Treacher Collins syndrome gene." Genome Res **7**(3): 223-234.

El-Gebali, S., J. Mistry, A. Bateman, S. R. Eddy, A. Luciani, S. C. Potter, M. Qureshi, L. J. Richardson, G. A. Salazar, A. Smart, E. L. L. Sonnhammer, L. Hirsh, L. Paladin, D. Piovesan, S. C. E. Tosatto and R. D. Finn (2019). "The Pfam protein families database in 2019." Nucleic Acids Res **47**(D1): D427-D432.

Marsh, K. L., J. Dixon and M. J. Dixon (1998). "Mutations in the Treacher Collins syndrome gene lead to mislocalization of the nucleolar protein treacle." Hum Mol Genet **7**(11): 1795-1800.

Sakai, D. and P. A. Trainor (2009). "Treacher Collins syndrome: unmasking the role of Tcof1/treacle." Int J Biochem Cell Biol **41**(6): 1229-1232.

Splendore, A., E. W. Jabs, T. M. Felix and M. R. Passos-Bueno (2003). "Parental origin of mutations in sporadic cases of Treacher Collins syndrome." Eur J Hum Genet **11**(9): 718-722.

Teber, O. A., G. Gillessen-Kaesbach, S. Fischer, S. Bohringer, B. Albrecht, A. Albert, M. Arslan-Kirchner, E. Haan, M. Hagedorn-Greiwe, C. Hammans, W. Henn, G. K. Hinkel, R. Konig, E. Kunstmann, J. Kunze, L. M. Neumann, E. C. Prott, A. Rauch, H. D. Rott, H. Seidel, S. Spranger, M. Sprengel, B. Zoll, D. R. Lohmann and D. Wieczorek (2004). "Genotyping in 46 patients with tentative diagnosis of Treacher Collins syndrome revealed unexpected phenotypic variation." Eur J Hum Genet **12**(11): 879-890.

UniProt, C. (2019). "UniProt: a worldwide hub of protein knowledge." Nucleic Acids Res **47**(D1): D506-D515.

Wang, S., W. Li, S. Liu and J. Xu (2016). "RaptorX-Property: a web server for protein structure property prediction." Nucleic Acids Research **44**(W1): W430-W435.

Wise, C. A., L. C. Chiang, W. A. Paznekas, M. Sharma, M. M. Musy, J. A. Ashley, M. Lovett and E. W. Jabs (1997). "TCOF1 gene encodes a putative nucleolar phosphoprotein that exhibits mutations in Treacher Collins Syndrome throughout its coding region." Proc Natl Acad Sci U S A **94**(7): 3110-3115.

**Protein:** TCF12; Transcription factor 12

**Function:** Member of the basic helix-loop-helix (bHLH) E-protein family. It is expressed in many tissues, such as bone, skeletal muscle, thymus, B- and T cells, and may participate in regulation of lineage-specific gene expression through the formation of heterodimers with other bHLH E-proteins such as TWIST1 (Paumard-Hernandez, Berges-Soria et al. 2015).

**Mutation:** p.Pro214Arg

**Structural analysis**: The protein is composed mainly of disordered and low complexity regions and contains a bHLH domain that ranges from residues 578 to 631. Two activation domains and a Rep domain have also been identified previously (Aronheim, Shiran et al. 1993, Markus, Du et al. 2002).

**Predicted effect:** Pro214 is found in between the activation domains 1 and 2, in a region of unknown significance predicted to be disordered by RaptorX (Wang, Li et al. 2016). Currently there is not enough structural information available to produce a reliable model of the mutated region.

A number of mutations have been previously identified in TCF12 from individuals with craniosynostosis, including the missense mutations L483R (Paumard-Hernandez, Berges-Soria et al. 2015), L600P (Sharma, Fenwick et al. 2013) and Q614E (Sharma, Fenwick et al. 2013)

**References**

Aronheim, A., R. Shiran, A. Rosen and M. D. Walker (1993). "The E2A gene product contains two separable and functionally distinct transcription activation domains." Proc Natl Acad Sci U S A **90**(17): 8063-8067.

Markus, M., Z. Du and R. Benezra (2002). "Enhancer-specific modulation of E protein activity." J Biol Chem **277**(8): 6469-6477.

Paumard-Hernandez, B., J. Berges-Soria, E. Barroso, C. I. Rivera-Pedroza, V. Perez-Carrizosa, S. Benito-Sanz, E. Lopez-Messa, F. Santos, R. Garcia, II, A. Romance, J. M. Ballesta-Martinez, V. Lopez-Gonzalez, A. Campos-Barros, J. Cruz, E. Guillen-Navarro, J. Sanchez Del Pozo, P. Lapunzina, S. Garcia-Minaur and K. E. Heath (2015). "Expanding the mutation spectrum in 182 Spanish probands with craniosynostosis: identification and characterization of novel TCF12 variants." Eur J Hum Genet **23**(7): 907-914.

Sharma, V. P., A. L. Fenwick, M. S. Brockop, S. J. McGowan, J. A. Goos, A. J. Hoogeboom, A. F. Brady, N. O. Jeelani, S. A. Lynch, J. B. Mulliken, D. J. Murray, J. M. Phipps, E. Sweeney, S. E. Tomkins, L. C. Wilson, S. Bennett, R. J. Cornall, J. Broxholme, A. Kanapin, C. Whole-Genome Sequences, D. Johnson, S. A. Wall, P. J. van der Spek, I. M. Mathijssen, R. E. Maxson, S. R. Twigg and A. O. Wilkie (2013). "Mutations in TCF12, encoding a basic helix-loop-helix partner of TWIST1, are a frequent cause of coronal craniosynostosis." Nat Genet **45**(3): 304-307.

Wang, S., W. Li, S. Liu and J. Xu (2016). "RaptorX-Property: a web server for protein structure property prediction." Nucleic Acids Res **44**(W1): W430-435.
